# A Compound of Operative Gynecology

**Published:** 1906-09

**Authors:** 


### BOOK REVIEWS.

A Compend of Operative Gynecology. Based on Lectures in the Course of Operative Gynecology on the Cadaver at the New York Postgraduate Medical School and Hospital, Delivered by William Seaman Bainbridge, M.D., Adjunct Professor of Operative Gynecology on the Cadaver, New York Postgraduate Medical School and Hospital; Consulting Gynecologist, St. Mary's Hospital, Jamaica, L. I.; Consulting Gynecologist to St. Andrew's Convalescent Hospital, New York, etc. Compiled, with additional notes, in collaboration with Harold D. Meeker, M.D., Instructor in Operative Gynecology on the Cadaver, New York Postgraduate Medical School and Hospital; Assistant, Department of Gynecology, Vanderbilt Clinic, College of Physicians and Surgeons, New York. 12mo. cloth, 76 pages, price $1.00, net. The Grafton Press, publishers, New York City.

This work, while particularly suited to the needs of postgraduate students operating on the cadaver, will be found of distinct value to the busy gynecologist. All gynecological operations of merit, with their latest modifications, are described concisely, but with sufficient detail to make the work decidedly practical. A number of original points of worth in the operative technic are embodied in the text.

A distinctive feature is the chapter on Exploration of the Viscera. Considering the gynecologist as an abdominal surgeon who should be able to cope with any intra-abdominal condition which may be encountered, the normal appearance and relations of abdominal viscera are briefly described in order to facilitate the familiarity essential to successful surgery.

In the chapter on Miscellaneous Points are many admirable suggestions of a practical nature which are timely and valuable.

The work is based on Dr. Bainbridge's lectures given in the course of Operative Gynecology on the Cadaver at the New York Postgraduate Medical School and Hospital.

It is a book that can be recommended.

